# Does cystoscopy method affect the investigation of bladder pain syndrome/interstitial cystitis?

**DOI:** 10.1007/s00192-020-04512-0

**Published:** 2020-09-21

**Authors:** Visha K. Tailor, Ellen Morris, Alka A. Bhide, Ruwan Fernando, Guiseppe A. Digesu, Vik Khullar

**Affiliations:** 1 Department of Urogynaecology, Imperial College NHS Trust, St Marys Hospital, 4th Floor Mary Stanford Building, London, W2 1NY UK; 2 Imperial College School of Medicine, Kensington, London, SW7 2DD UK

**Keywords:** Bladder biopsy, Bladder pain syndrome/interstitial cystitis, Cystoscopy, Bladder mastocytosis

## Abstract

**Introduction and hypothesis:**

Cystoscopic investigation to identify associated histological findings of increased mast cells in the detrusor muscle has been recommended by the European Society for the Study of Bladder Pain Syndrome (ESSIC) in the investigation of bladder pain syndrome/interstitial cystitis (BPS/IC). The aim of this study was to identify if the cystoscopy approach impacts the biopsy results when investigating women presenting with symptoms of BPS/IC.

**Methods:**

We performed a single-centre retrospective analysis of 300 bladder biopsy reports from 2015 to 2018 from women undergoing cystoscopy for BPS/IC. Biopsies obtained using closed cup forceps through a flexible (FC) or rigid cystoscope (RC) were compared.

**Results:**

Fifty-eight FC biopsies were compared with 242 RC biopsies. FC biopsies had a smaller mean diameter (1.6 mm vs 2.9 mm *p* < 0.01) and volume (4.1 mm3 vs 9.6 mm^3^ *p* < 0.001) compared with RC biopsies. There was no significant difference in the histological depth of sampling to the muscularis propria. A total of 292 samples had CD117 immunohistochemical staining for mast cell count (MCC) analysis. The MCC/mm^2^ was significantly lower in FC biopsies (*p* < 0.01). Sixteen percent of FC samples compared with 60% of RC samples had a high MCC >28/mm^2^ (*p* < 0.01). There was no significant difference in positive microbiology culture between FC (21%) and RC (28%) sampling.

**Conclusion:**

Rigid and flexible cystoscopy can be used to investigate BPS/IC as recommended by international societies. However, the biopsy method impacts the mast cell count analysis, which can influence diagnosis and management. Therefore, RC would be the optimal investigation.

## Introduction

Bladder pain is defined as suprapubic or retropubic pain, pressure, or discomfort, related to the bladder. It often increases with bladder filling and may persist or be relieved after voiding [1]. When bladder pain presents with persistent lower urinary tract symptoms such as urinary frequency, urgency or nocturia, in the absence of identifiable pathology or causes, the diagnosis of bladder pain syndrome (BPS) is given [2]. This is a chronic condition with much to unravel about its aetiology and prognosis. Many international societies have developed guidelines to aid clinicians in the management of this complex condition; however, international consensus for all aspects has not yet been reached. Symptoms of BPS must be present for a minimum of 6 weeks (American Urological Association [3]) to 6 months (European Society for the Study of Bladder Pain Syndrome (ESSIC) [4]) before a clinical diagnosis is made.

Bladder pain syndrome is described alongside interstitial cystitis (IC), standardised as BPS/IC. IC alone is a controversial term, it is used for reimbursement in some healthcare systems, but is currently mostly considered synonymous with BPS. There is discussion amongst the scientific community that IC could represent a separate phenotype or subtype under the overall umbrella diagnosis of BPS [5]. The characteristic feature of IC is the finding of a typical bladder lesion as originally described by Hunner in 1915; this is the appearance of patches of red mucosa exhibiting small vessels radiating to a central pale scar [6]. This Hunner’s lesion can rupture with increasing bladder distension, demonstrating petechial bleeding or bleeding in a waterfall manner at the margins of the lesion. Following distension, a typical bullous oedema can develop [4].

The diagnosis of BPS is typically a clinical one. Cystoscopy is recommended to investigate alternative causes of the symptoms such as bladder carcinoma, bladder stones or endometriosis [4, 5]. However, ESSIC and the ICS standardisation working group specifically recommend investigation by cystoscopy to identify Hunner’s lesions and further classify BPS/IC [4, 7]. ESSIC describes a standardised method using rigid cystoscopy (RC) with hydrodistension to evaluate patients with BPS/IC. Owing to the anticipated discomfort of the procedure, spinal or general anaesthesia is stipulated [4]. The use of a rigid cystoscope is thought to facilitate adequate biopsy sampling and also allows for coagulation after biopsy. Flexible cystoscopy (FC) in an office setting for the initial assessment of BPS/IC is also practiced [8, 9]. This is reported to be a well-tolerated procedure carried out in an office setting, making it cost effective. Associated cystoscopic findings include petechiae, glomerulations and increased bladder vascularity. These findings can also be observed in women with no history of bladder pain and are therefore alone not diagnostic of BPS/IC [4, 7, 10].

The aetiology of BPS/IC is complex and heterogeneous, with mast cells (MCs) thought to play a role in the development and persistence of pain associated through a self-perpetuating cycle of MC activation and recruitment. The ESSIC recommends three bladder biopsies to be taken during cystoscopy and assessed for increased mast cell counts (MCCs) or MCs in the detrusor muscle [4]. At present, only the ESSIC identifies MC infiltrates within the detrusor muscle as a diagnostic criterion for BPS/IC [4]. There are otherwise no internationally recommended diagnostic histopathological findings for BPS/IC. A MCC >28 MCs/mm^2^ is considered to indicate significant mastocytosis [4]. Other associated histological findings include: chronic inflammatory infiltrate, denuded epithelium, submucosal inflammation, granulation tissue, intrafascicular fibrosis and MC infiltration [4, 11].

Mast cells play a role in both innate immunity and neuroinflammatory conditions [12]. Urothelial damage or dysfunction causes release of neuropeptides and neurotransmitters that recruit and activate MCs [13]. Increased permeability of the urothelium also allows an influx of potassium ions that in turn leads to sensory afferent nerve upregulation, tissue damage and MC activation [13]. Activation of MCs through these stimuli leads to degranulation and release of vasoactive and inflammatory mediators that further promote inflammation, MC activation and fibrosis [8].

The finding of a raised MC may play a role in clinical phenotyping, as well as providing prognostic value, which can aid counselling of patients diagnosed with BPS/IC. A retrospective Danish study of BPS/IC patients by Richter et al. [9] found that 53% of the study group had detrusor mastocytosis. Detrusor mastocytosis correlated with more frequent and advanced treatment, including pain clinic referral, high-dose steroids and surgery with cystoplasty or urinary diversion. Globally, there is varying emphasis placed on the role of cystoscopy and bladder biopsy in the diagnosis of BPS/IC, with Asia and Europe prioritising this investigation compared with North America [5].

The aim of this study was to identify if the cystoscopy approach has an impact on biopsy quality. We compared bladder biopsy results taken using closed-cup forceps using either a flexible cystoscope (FC) or rigid cystoscope (RC). A null hypothesis of no differences between the two biopsy types is proposed.

## Materials and methods

We performed a single-centre retrospective analysis of histology and microbiology bladder biopsy reports from 2015 to 2018. This study reports the results of a service evaluation/audit for which local approval was obtained (GRM_025). Three hundred women with BPS/IC were identified through electronic operation notes as the indication for cystoscopy.

Bladder biopsies were obtained using closed-cup forceps through either a flexible or a rigid cystoscope by an experienced urogynaecologist. FC was carried out in an office setting following administration of 2% lidocaine hydrochloride gel (Instillagel®). The bladder was distended with normal saline, most commonly to comfortable capacity to allow diagnostic cystoscopy prior to biopsy sampling. Emptying and refilling of the bladder was not carried out prior to biopsy.

Rigid cystoscopy was carried out under general anaesthesia as recommended by ESSIC [4]. Bladder biopsy was obtained following initial distention, inspection and subsequent re-filling of the bladder. Normal saline was used as the distension medium. Diathermy to the biopsy site was carried out to achieve haemostasis.

One or two biopsy specimens were taken. One sample was immediately fixed in 10% formalin and sent for histology and the other for microbiology assessment. Analysis of MCC was carried out using CD117 immunohistochemical staining. Counting was carried out using a graticule to produce one MCC per square millimetre. Reports were compared.

Statistical analysis was carried out using SPSS 25 software. Unless stated, independent *t* test for continuous variables and Chi-squared test for independence for ordinal variables was used.

## Results

A total of 300 women clinically diagnosed with BPS/IC were identified, with a mean age of 46 years (range 17–90 years). Fifty-eight women underwent FC with biopsy compared with 242 women who underwent RC with biopsy. Women undergoing RC were younger than women having FC (44.5 years vs 53.2 years, mean difference 8.6 ± 2.4 years, *p* < 0.001). At cystoscopy, none of the women in this study was found to have Hunner’s lesions.

Four overall histopathology diagnoses were reported by the laboratory (Table 1): normal, chronic inflammation, chronic cystitis, and interstitial cystitis. There was no difference in the reporting frequency of the overall diagnosis between the FC and RC biopsy groups (Fisher’s exact test, *p* = 0.072).

**Table 1:** There was no significant difference in the frequency of the histopathological diagnosis given depending on the biopsy method (Fisher’s exact test, *p* > 0.072)

Diagnosis	Rigid cystoscopy, *n* (%)	Flexible cystoscopy, *n* (%)
Normal	8 (3.3)	6 (10.3)
Chronic inflammation	119 (49.2)	32 (55.2)
Chronic cystitis	84 (34.7)	16 (27.6)
Interstitial cystitis	31 (12.8)	4 (6.9)

Flexible cystoscopy biopsies had a smaller mean width, height, thickness (1.67 × 1.38 × 1.17 mm vs 2.85 × 1.89 × 1.49 mm, *p* < 0.001) and volume (4.1 mm^3^ vs 9.6 mm^3^, *p* < 0.001) compared with RC biopsies. Thirty biopsies had a volume ≤ 1 mm^3^, 73% of which were obtained using FC. Where reported, although there was a trend towards a greater depth of sampling using RC, there was no significant difference in the histological depth of sampling to the muscularis propria between FC (14 out of 52, 27%) and RC (82 out of 216, 38%) sampling methods.

A total of 292 samples had CD117 immunohistochemical staining for MMC analysis. Two hundred and sixty-four had MCs/mm^2^ reported; the remaining were reported as higher or lower than 28 MCs/mm^2^. Four out of 57 FC samples (7%) were reported to be inadequately sized to carry out an MCC. There was no correlation between the age of the patient and the MCC/mm^2^ (r_s_ = −0.02).

Flexible cystoscopy biopsies had a mean MCC of 21 MCs/mm^2^, with a range of 2–90 MCs/mm^2^. RC biopsies had a mean MCC of 36 MCs/mm^2^, with a range of 6–100 MCs/mm^2^. The mean MCC was significantly lower in FC biopsies than in the RC biopsy analysis (*p* < 0.01, independent samples *t* test).

Overall, 51% of biopsies were reported to have a high MCC >28/mm^2^, of which 90% were obtained using RC. Sixteen percent of FC samples compared with 60% of RC samples had a high MCC (*p* < 0.001, Chi-squared test of independence).

One-way ANOVA was carried out to explore the relationship between the histopathological diagnosis and the MCC. Six outlier results were identified, as assessed by inspection of box plots, and included in the analysis. MCCs were normally distributed for the normal, chronic cystitis and interstitial cystitis groups, as assessed by the Shapiro–Wilk test (*p* > 0.05). The mean MCC between the different histopathological diagnoses were statistically different (Welch *F* (3, 49.4) = 30.8, *p* < 0.001). Further analysis demonstrated a significant difference between all groups (Games–Howell test, *p* < 0.01) except between the chronic cystitis and the interstitial cystitis diagnosis (Games–Howell test, *p* = 0.09; Table 2).

**Table 2:** Mean mast cell count (MCC)/mm^2^ reported for each histopathology diagnosis. There was a significant difference between the overall mean MCC for each histopathology diagnosis (*p* < 0.01) except between the chronic cystitis and the interstitial cystitis diagnosis (*p* = 0.09). Despite a trend towards higher MCC from rigid cystoscopy biopsies, there was no significant differences between the results depending on the biopsy method

Diagnosis	Mean MCC/mm^2^	MCC by rigid cystoscopy/mm^2^	MCC by flexible cystoscopy/mm^2^
Normal	17	22	7
Chronic inflammation	28	*29*	20
Chronic cystitis	*40*	*42*	23
Interstitial cystitis	*48*	*47*	*50*

Cells in *italics* represent results that can have an impact on clinical management

There was a positive correlation between increasing MCCs with increasing inflammation severity graded as mild, moderate or severe (*r*_*s*_ = 0.4101, *p* = 0.01). One-way ANOVA analysis found a significant difference between the mean MCC and the severity of the inflammation or cystitis reported (Welch *F* (3, 34.4) = 22.9, *p* < 0.001; Table 3). There were significant differences between normal, mild, moderate, and severe inflammation (Games–Howell test, *p* < 0.01) except between the moderate and severe inflammation groups (*p* = 0.81). The mean MCC for biopsies demonstrating any degree of inflammation signified significant mastocytosis except for severe inflammation reported on 2 biopsies obtained by FC.

**Table 3:** Mean mast cell count (MCC)/mm^2^ reported for each degree of inflammation severity reported. There was a significant difference between the overall mean MCC for each degree of severity (*p* < 0.01) except between moderate and severe inflammation (*p* = 0.81). Despite a trend towards higher MCC from rigid cystoscopy biopsies, there was no significant differences between the results depending on the biopsy method

Severity of inflammation	Mean MCC/mm^2^	MCC by rigid cystoscopy/mm^2^	MCC by flexible cystoscopy/mm^2^
No inflammation	16.5	22.4	7.6
Mild	*33.5*	*34.5*	*29.1*
Moderate	*40.3*	*41.6*	*34*
Severe	*42.2*	*42.2*	< 28 (*n* = 2)^a^

Cells in *italics* represent results that can have an impact on clinical management

^a^Quantified MCC was not reported for the flexible cystoscopy biopsies demonstrating severe inflammation

Univariate analysis was carried out to assess the impact of the biopsy method on the MCC for each histopathological diagnosis and severity of inflammation reported. No significant differences were identified, despite a trend of lower reported FC biopsy MCC.

In 238 a second biopsy was sent for microbiology culture. There was no significant difference in positive culture between FC (19.5%) and RC (29%) sampling (*p* = 0.218).

## Discussion

The results of this study support the use of both RC and FC to investigate BPS/IC. The biopsy method does not appear to have an impact on the overall histopathological diagnosis reported or the ability to carry out a microbiology culture. However, the biopsy method appears to have an impact on the MCC analysis, which can influence rates of BPS/IC diagnosis and management. Higher MCCs were associated with a higher degree of reported inflammation (moderate and severe).

The diagnostic usefulness of cystoscopy for BPS/IC has been questioned in the past. However, it is important to exclude alternative causes of pain, as well as to identify if Hunner’s lesions are present, as the clinical management will be guided accordingly. Effective treatment has been described specifically for Hunner’s lesions, including resection, ablation or electrocoagulation of the identified lesion [14, 15]. BPS/IC with Hunner’s lesions does not present with a distinct bladder-centric clinical phenotype [16]. Therefore, cystoscopic evaluation is required to distinguish this clinical group.

Flexible cystoscopy without general anaesthetic is a common cystoscopic approach for most urological investigations and interventions. It is considered to be cost effective as it can be carried out in the office setting. Studies have shown that both male and female patients tolerate FC with local anaesthesia better than RC with local anaesthesia, particularly for a first cystoscopy procedure [17, 18]. That said, there are studies demonstrating good tolerability of diagnostic RC in women [19], as well as for removal of double J-stents with the addition of diclofenac and/or intraurethral lignocaine to reduce pain [20].

In the investigation of BPS/IC, the concern would be that cystoscopy with local anaesthesia may be limited by pain. The use of FC specifically could make taking multiple biopsies an arduous task and biopsy samples may be inadequate in size or distorted by collection to adequately complete the MCC analysis. Seven percent of biopsy samples collected by FC were too small to process.

Rigid cystoscopy with hydrodistension is recommended by ESSIC for the evaluation of BPS/IC symptoms [4]. Whilst the evidence is inconsistent, there are also studies reporting the therapeutic benefits of this procedure. The evidence is not robust or strong enough to recommend this practice widely, but some practitioners will value this added perceived benefit of RC over FC methods [21]. There is also the additional benefit of determining the anaesthetic bladder capacity if RC with general anaesthesia is performed. Low bladder capacity can be associated with more severe symptom severity and Hunner’s lesions [11, 22, 23]. Urodynamic studies have shown that the maximum bladder capacity can be increased in women with bladder pain following administration of 20 ml of 2% alkalinised lidocaine to the bladder for 20 min [24]. Pain and the use of local anaesthetic gel may therefore have an impact on the accuracy of bladder capacity if determined by FC.

Doiron et al. describe the use of FC to successfully identify Hunner’s lesions without hydrodistension or general anaesthesia in their study of 359 women with BPS/IC [16]. Forty-four were found to have a Hunner’s lesion, described as a “circumscribed, reddened mucosal area with small vessels radiating towards a central scar, with a fibrin deposit or coagulum attached”. The team do not report if bladder biopsy was obtained or further management for the 315 patients with no Hunner’s lesions. RC with local anaesthesia has not been formally evaluated for the BPS/IC population; however, it could remain a considered option if general anaesthesia were inappropriate. A technique of two coughs to improve detrusor sampling using FC has been described to facilitate the tolerability of biopsy and may be applicable to the RC if used with local anaesthesia [25].

Our study demonstrates that biopsies obtained using RC are larger in size, and although there was a trend towards better sampling of the detrusor with RC (38% vs 27%), no significant difference was found. Previous studies emphasise that a higher MCC in the detrusor is more specific for BPS/IC than MCCs in the mucosa [16, 26, 27]. Multiple biopsy sampling as recommended by ESSIC may facilitate adequate sampling of the detrusor muscle [4]. It can however be technically difficult to obtain adequate biopsy to allow consistent evaluation of the detrusor muscle whilst balancing the risks of bladder perforation and patient comfort if awake. Gamper et al. studied the MCC from bladder biopsies obtained by RC and cold-cup forceps under general anaesthesia [28]. As with our experience, sampling of the detrusor is not always consistent. Thirty percent of their biopsies met the ESSIC recommendation of greater than 1 mm^2^ of detrusor muscle. All other MCC results were extrapolated to 1 mm^2^, where the biopsy sample was too small. Both Gamper et al. and Malik et al. report subepithelial and detrusor mastocytosis in BPS/IC [28, 29]. Therefore, even in the absence of complete detrusor sampling, the MCC can contribute to the diagnostic evidence of BPS/IC.

Overall, 51% were found to have a raised biopsy MCC that would influence their ongoing clinical management. Activation of MCs leads to degranulation and release of many inflammatory mediators. Release of histamine from MCs is considered to be related to inflammation and hypersensitivity of the bladder. Shan et al. studied the expression of histamine receptors within the bladder of women with BPS/IC [30] and identified increased expression of all four histamine receptors in the BPS/IC group compared with the control group. Although we report no treatment outcomes for this study, the authors would explore the association of bladder mastocytosis with a possible diagnosis of MC activation syndrome and would consider treatment with a trial of diet restrictions to avoid MC-liberating foods in combination with MC-stabilising or suppression therapy [31, 32]. Lower MCCs obtained by FC may not reflect the true result and would therefore have an impact on clinical management. For example, the women found to have severe inflammation reported on biopsy obtained by FC had low MCCs reported (Table 3). This could represent an alternative aetiology or inadequate sampling for MCC analysis.

Only 10% of the biopsy reports with a high MCC were obtained using FC. There was also a greater trend towards normal biopsy reporting, with normal MCCs (10% vs 3%) for FC results. These results may reflect the fact that FC produces a smaller biopsy. Alternatively, these results may reflect the limitations of our study. Choice of cystoscopy method in our unit is based on patient preference (for example, to avoid a general anaesthetic) or if there were significant comorbidities, which could introduce selection bias to our results. Patients undergoing FC were older than those undergoing RC. We did not have complete demographic data to understand if this was related to underlying health or if it reflected a milder symptom profile. Those with more severe symptoms may have wanted to avoid a potentially painful procedure or be guided towards a RC, as per ESSIC guidance [4]. As a result, we do have two study groups of different sizes, which does make this a challenging comparison of results.

The histopathology reports were written by two pathologists for this study. There was inconsistent reporting of bladder biopsy size and layers sampled with 11% (*n* = 32) not being reported. Nine percent (*n* = 28) had reporting of the MCC as high or low, with no formal MCC/mm^2^ reported. Our results demonstrated that any reported inflammation given in the overall diagnosis was associated with a mean MCC ≥28/mm^2^ (Table 2). However, to our knowledge, there is no formal guidance distinguishing chronic inflammation from chronic cystitis or interstitial cystitis, or guidance to report the severity of the inflammation. It can be argued that they could all be associated with BPS/IC. The associated histopathological findings of BPS/IC are well described, with the ESSIC recommending inflammatory infiltrates and/or detrusor mastocytosis and/or granulation tissue and/or intrafascicular fibrosis as “positive” histology for BPS/IC [4]. Thirty-five biopsies in our study were reported with a histopathology diagnosis of IC; however, there are no diagnostic histopathological features of BPS/IC. Although the biopsy reporting may reflect the experience of the histopathologist, the lack of standardised reporting does limit the comparisons that can be made between patients. Unfortunately, this limitation is one that has also had an impact on previous histopathological studies [11].

In our study, the laboratory reported the MCC/mm^2^ using a CD117 immunohistochemical stain for subepithelial tissue for most biopsies. This method targets a MC transmembrane tyrosine kinase receptor, which is considered to be a sensitive and accurate method of identifying MCs. Although we have utilised the cut-off for mastocytosis of >28 MCs/mm^2^, this value has been established for biopsies stained with the Leder stain (naphthol esterase cytochemical stain) [4, 27]. Leder staining identifies intact MCs and diminishes if the MC is activated, leading to degranulation [10]. CD117 immunohistochemical stain does not distinguish between MCs that are degranulated or those that are not [33]. Further research establishing CD117 immunohistochemical staining and the parameters for bladder mastocytosis are therefore urgently required, as this method of staining is already widely used in the investigation of alternative MC-related diagnoses, as well as for bladder biopsy MCC analysis [11, 33].

## Conclusion

To our knowledge, this is the first study reporting differences in bladder biopsies between two cystoscopy methods. There is established scientific evidence to conclude that the MCC contributes to the diagnostic evidence of BPS/IC. Our study results favour the use of RC over the use of FC to obtain bladder biopsies for MCC analysis, as advised by ESSIC [4]. There is the potential for inadequate sampling and under-diagnosis of mastocytosis from FC biopsies. Over time, we hope for further international consensus and guidance in the investigation of BPS/IC, particularly to standardise and update MCC analysis methods and histopathology reporting.

## Abbreviations

BPS/IC: Bladder pain syndrome/interstitial cystitis
FC: Flexible cystoscopy
MC: Mast cell
MCC: Mast cell count
RC: Rigid cystoscopy
